# Acute and sub-acute toxicological studies on the ethanol leaf extract of *Psidium guajava* in experimental mice

**DOI:** 10.11604/pamj.2026.53.49.49406

**Published:** 2026-02-03

**Authors:** Getahun Tsegaye Dibaba, Abebaye Aragaw Leminie, Wossene Habtu Tadesse, Worku Gemechu Lemmi, Samuel Woldekidan Hirpesa, Moti Sori Reje, Tsegay Beyene Weldemariam, Mekoya Mengistu Dabulo, Beza Tasew Degefu, Menberework Chanyalew, Tesfaye Tolessa Dugul

**Affiliations:** 1 School of Medicine, College of Health Sciences, Addis Ababa University, Addis Ababa, Ethiopia,; 2 National Clinical Chemistry Reference Laboratories, Ethiopia Public Health Institute, Addis Ababa, Ethiopia,; 3 Traditional and Modern Medicine Directorates, Armauer Hansen Research Institute, Addis Ababa, Ethiopia,; 4 Non-communicable Disease Division, Armauer Hansen Research Institute, Addis Ababa, Ethiopia,; 5 St. Joseph University in Tanzania (SJUIT), Dar es Salaam, Tanzania

**Keywords:** *: Psidium guajava*, ethanolic extract, acute toxicity, sub-acute toxicity, mice

## Abstract

**Introduction:**

Psidium guajava (guava) is an important medicinal plant used for gastrointestinal disorders, diabetes, and inflammation, primarily due to its bioactive compounds including flavonoids and tannins. To ensure safety for human use, rigorous preclinical toxicity assessments are essential. The purpose of this study was to assess the safety of ethanol extract of Psidium guajava (EEPG) by acute and subacute toxicity tests.

**Methods:**

Organization for Economic Co-operation and Development (OECD) 425 recommendations were used for the acute toxicity investigation in mice, while OECD 407 guidelines were used for the sub-acute toxicity study in mice. Mice were given oral doses of 2000 mg/kg/ body weight in the acute toxicity trial. They were then monitored individually for the first four hours, then for 24 hours, then at least once a day for 14 days. Ethanol extract of Psidium guajava (EEPG) was administered orally to male and female mice for 28 days at dosages of 250 mg/kg, 500 mg/kg, and 1000 mg/kg /body weight, respectively, in the subacute toxicity tests. Over the course of the experiment, general behavior, negative impacts, and mortality were noted. Hematological and biochemical markers, body weight, organ weight, and histopathological alterations were assessed.

**Results:**

in acute toxicity during the observation period, the mice examined showed no evidence of acute toxicity or mortality at the limit dosages of 2000 mg/kg/body weight. The findings of sub-acute toxicity testing revealed no anomalies associated with treatment in terms of biochemical, hematological and histopathology markers at the maximum dose of 1000 mg/kg /body weight.

**Conclusion:**

the substance showed a favorable safety profile, with no acute and subacute toxicity or mortality at 2000 mg/kg and no treatment-related adverse effects at 1000 mg/kg/body weight/day in mice.

## Introduction

Natural products derived from herbs, animals, and minerals constitute a historically unparalleled and chemically diverse library for drug discovery. Iconic medications such as the antimalarial artemisinin (from *Artemisia annua*), the cardiac glycoside digoxin (from *Digitalis purpurea*), and the analgesic morphine (from *Papaver somniferum*) underscore how these resources have long served as indispensable lead compounds for developing novel therapeutics [1]. Despite the sophisticated techniques of modern pharmacotherapy, this traditional knowledge base remains a crucial and validated foundation for contemporary drug discovery, with a significant portion of current pharmaceuticals owing their origins to natural product research [1]. The sustained and growing public interest in herbal remedies is largely driven by the widespread perception of their being “natural” and therefore inherently safer than synthetic drugs [2]. However, this perception creates a critical paradox: a commensurate body of rigorous scientific evidence on their safety profiles [3] does not consistently match the global increase in consumption. All bioactive compounds, irrespective of origin, carry inherent risk potential; necessitating those herbal products is subjected to the same systematic toxicological scrutiny as conventional pharmaceuticals. In response to this need for standardization, international regulatory bodies have developed specific frameworks for safety evaluation. The World Health Organization's Uppsala Monitoring Centre (UMC) plays a key role in pharmacovigilance by collating global data on adverse drug reactions, including those associated with herbal products [4].

Concurrently, the Organization for Economic Co-operation and Development (OECD) provides the definitive experimental guidelines, such as Test No. 425 (Acute oral toxicity) and Test No. 407 (Repeated dose 28-day oral toxicity), that govern the conduct of standardized toxicity tests [5]. These tests, spanning acute and subacute, are designed to investigate specific adverse endpoints and are fundamental for establishing the no observed adverse effect level (NOAEL), a critical datum for informing safe dosing in subsequent clinical trials [4-6]. *Psidium guajava* leaf (guava), a member of the Myrtaceae family, exemplifies a plant at the intersection of traditional valorization and modern scientific inquiry. Local name in Ethiopia is “zeituna” and Native to Central and South America and now cultivated pan tropically, its leaves are a repository of bioactive phytochemicals, including flavonoids (e.g., quercetin), tannins, and essential oils, which are credited for their antioxidant, antimicrobial, and anti-inflammatory properties demonstrated in preliminary studies [6,7]. In ethno-medical practices across diverse cultures, including in Ethiopia, guava leaf preparations are commonly employed to manage conditions such as diarrheal diseases, diabetes, and malaria [7]. Although it is widespread traditional use in Ethiopia and has a promising phytochemical portfolio, a comprehensive toxicological profile of standardized *Psidium guajava* leaf extracts remains inadequately documented, particularly within the local scientific context. Therefore, the primary aim of this study is to address this gap by providing essential pre-clinical safety data. The specific objectives are to systematically evaluate the toxicological profile of a characterized ethanol leaf extract of *Psidium guajava* in a murine model, to determine the acute oral median lethal dose (LD_50_), to assess sub-acute oral toxicity through detailed hematological analysis, serum biochemical panels (with a focus on liver and kidney function markers), and histopathological examination of vital organs.

## Methods

**Collection and extraction of plant material:** in September 2024, fresh *P. guajava* leaves were acquired from the Seka district in the Jimma Zone of the Oromia Region, Ethiopia. The plant samples were brought to the laboratory settings in the Traditional and Modern Medicine Directorates, Armauer Hansen Research Institute, Ethiopia and identified by comparison with herbarium specimens housed in the Department of Botany at Addis Ababa University. The scientific name *Psidium guajava* was confirmed, and a voucher sample (GT001) was authenticated by Melaku Wendafrash, a botanist at the Addis Ababa University Herbarium Center. The leaves were thoroughly washed to remove debris, shade-dried at room temperature (25-30°C) for 10 days, and ground into a fine powder using an electric grinder. The powder was then sieved through a 60-mesh sieve (0.25 mm pore size) [8]. For ethanol extraction, 100 g of the powdered leaves were macerated in 1200 mL of 70% ethanol (a 1: 12, w/v ratio) for 48 hours at room temperature with occasional stirring. This ratio and duration were based on preliminary optimization for maximizing the recovery of polar and non-polar phytochemicals. The mixture was then shaken for 2 hours on an orbital shaker (150 rpm), filtered through Whatman No. 1 filter paper, and concentrated under reduced pressure (40°C, 60 rpm) using a rotary evaporator [9]. Ethanol (70%) was selected for its high efficiency in extracting bioactive compounds such as flavonoids, alkaloids, and phenolic acids [10]. The resulting extract, with a yield of 17.25% ± 0.20%, was stored at -20°C in airtight amber vials to prevent degradation.

**Experimental animals:** Swiss albino mice were utilized for the toxicity assessments. Animals weighing 30-40 g and 10 weeks old were selected for the acute toxicity study, while both male and female mice were used for the subacute (28-day) investigation. All mice were housed under standard laboratory conditions, maintaining a temperature of 20-26°C with a 12-hour light/dark cycle, and were provided standard feed and water *ad libitum*. Prior to experimentation, the animals underwent a one-week acclimatization period. The study protocol referred to ethical approval from the Institutional Animal Ethics Committee (IAEC), and all procedures adhered to the guidelines of the Committee for the Purpose of Control and Supervision of Experiments on Animals (CPCSEA). In addition, ethical clearance was received from the Addis Ababa University, College of Health Sciences Institutional Review Board (Protocol No. 044/23/physio) [7,8].

**Euthanasia and sample collection:** upon completion of the experimental protocols, all animals were humanely euthanized via CO_2_ inhalation in accordance with the approved methods stipulated by the Addis Ababa University Ethical Review Board and international standards. The procedure employed a controlled, gradual fill rate (30-70% of the chamber volume per minute) as recommended by the American Veterinary Medical Association (AVMA) Guidelines [11] to minimize distress and ensure unconsciousness prior to potential nociception. This method was selected for its rapid anesthetic effect, cost-effectiveness, and minimal interference with post-mortem biochemical analyses. To reduce pre-euthanasia stress, animals remained in their home cages until the procedure. Chamber temperature and gas distribution were meticulously monitored to guarantee a uniform atmosphere. Death was confirmed by the absence of respiratory movement and cardiac activity [12]. Immediately following euthanasia, trained personnel performed cardiac puncture to obtain sufficient volumes of uncontaminated blood, thereby preventing stress-related artifactual changes in biochemical parameters. Vital organs, including the liver, kidney, and heart, were subsequently excised promptly to prevent autolysis and preserve morphological integrity for subsequent histopathological examination [13]. All procedures strictly adhered to the OECD Guidelines for the Testing of Chemicals and were conducted under the oversight of Addis Ababa University's Institutional Ethical Guidelines (Protocol number 044/24/physio).

**Acute toxicity testing:** the acute oral toxicity test was conducted in accordance with the Organization for Economic Co-operation and Development (OECD) Test Guideline 425 [14]. Ten Swiss albino mice (five males and five females) were fasted for six hours prior to administration. Each animal was weighed, and a single oral dose of the *P. guajava* leaf extract was administered via gavage at a concentration of 2000 mg/kg/ body weight. Following administration, food was withheld for an additional four hours. During this immediate post-administration period, each mouse was individually monitored for signs of acute toxicity, including changes in locomotor activity, aggressive behavior, piloerection, facial expressions, and responsiveness to tactile or auditory stimuli. Subsequently, all animals were observed daily for a period of 14 days to monitor for any delayed signs of morbidity or mortality. At the conclusion of the observation period, biological samples were collected from all animals following the standardized procedures described in the preceding section.

**Sub-acute toxicity studies:** a sub-acute toxicity study (28-day repeated oral dose) was conducted according to OECD Test Guideline 407 [15]. Forty (40) Swiss albino mice (30-40 g) were ten weeks of age. In brief, 40 Swiss albino mice were randomly divided into 4 groups of 10 animals each (05 males and 05 females in a separate cage). Group I served as the control and received a daily oral dose of the vehicle (1% carboxymethyl cellulose, CMC) at 10 ml/kg body weight. The treated groups received the *P. guajava* leaf extract (EEGP) suspended in the same vehicle at doses of 250 mg/kg (Group II), 500 mg/kg (Group III), and 1000 mg/kg/body weight (Group IV), administered orally at a constant volume of 10 ml/kg daily. Throughout the 28-day treatment period, all mice were observed twice daily for signs of morbidity and mortality. Any clinical symptoms were recorded, including their nature, time of onset, and duration. Individual body weights were measured prior to the first dose, at weekly intervals during treatment, and on the day of sacrifice.

**Hematological parameters:** using a fully automated hematology analyzer (KX21), the heparinized blood was utilized to measure hematological parameters such as hemoglobin, red blood cell count, white blood cell count, and platelet count.

**Biochemical parameters:** after the serum was extracted from non-heparinized blood, a semi-automatic biochemical analyzer (5010 Germany) was used to measure the serum's biochemical parameters, which included total cholesterol, creatinine, alanine aminotransferase (ALT), aspartate aminotransferase (AST), alkaline phosphatase (ALP), blood urea nitrogen (BUN), triglycerides, albumin, bilirubin, and total protein.

**Histopathology:** after blood collection on day 29, all the animals are euthanized for gross pathological evaluations of main internal organs. Organs such as the liver, kidney, and heart were obtained from all the animals for histopathology. The tissue was sent to the pathologist blindly to avoid bias. After being weighed and fixed in 10% neutral buffered formalin, the collected organs were cut into tissue slices that were five µm thick and stained with hematoxylin and eosin for histological analysis.

**Statistical analysis:** results are expressed as mean ± standard error of the mean (SEM). Differences between treatment groups and the control were analyzed using one-way ANOVA. Where the ANOVA indicated a significant overall effect, Dunnett's multiple comparisons test was applied to identify which specific groups differed from the control. Statistical significance was set at p < 0.05.

## Results

**Acute toxicity studies:** oral EEPG delivery at 2000 mg/kg did not cause any mouse fatalities or clinical toxicity symptoms in the toxicity investigation. Since neither of the tested doses showed any clinical symptoms of toxicity or fatality, the EEPG LD50 value was determined to be higher than 2000 mg/kg/body weight.

**Sub-acute toxicity studies:** during the four weeks of treatment, mice given oral doses of 250 mg/kg, 500 mg/kg, and 1000 mg/kg showed no evidence of treatment-related toxicity or mortality in either sex. The initial and ultimate body weights of the mice treated with EEPG and the control animals did not differ significantly (Table 1). Organ weight did not significantly differ between the control and EEPG-treated groups (Table 2). Table 3 provided a summary of the hematological profiles of the treatment and control groups. The findings showed that during the trial period, both the control and treatment groups' hematological parameters- including the total red blood cell count, total white blood cell count, platelet count, hemoglobin, hematocrit, and differential leukocyte count - were within the normal range. Table 4 displayed the biochemical parameter data for both treatment and control animals. When compared to control groups, biochemical parameters, including creatinine, urea, triglycerides, total cholesterol, total protein, albumin, aspartate aminotransferase (AST), alanine aminotransferase (ALT), alkaline phosphatase (ALP), and total bilirubin, did not significantly change following subacute EEPG administration. The hematological and biochemical parameters assessed in the control and EEPG-treated groups did not differ statistically significantly. Histopathology analyses of the heart, liver, and kidney in the control and high dose groups of our study showed no abnormalities. In mice, the extract's No-Observed Adverse Effect level (NOAEL) was calculated to be higher than 1000 mg/kg/day. Therefore, it can be said that EEPG is safe to use orally.

**Table 1:** effect of ethanol extract of *Psidium guajava* (EEPG) on body gain in mice-sub-acute

Treatment group	Body weight					
	Sex	Day 1	Day 7	Day 14	Day 21	Day 28
Group 1	Male (n=5)	31.00±2.00	33.20±1.17	36.00±1.58	39.00±1.58	42.00±3.74
	Female (n=5)	28.00±1.00	32.20±2.01	34.40±1.66	37.60±1.11	40.60±1.22
Group 2 250mg/kg/body weight	Male (n=5)	34.00±2.27	40.60±2.40	42.00±1.58	44.00±2.18	46.00±2.45
	Female(n=5)	31.40±1.21	34.40±1.12	38.40±2.02	40.30±2.11	42.40±2.00
Group 3 500mg/kg/body weight	Male (n=5)	40.00±1.12	42.00±1.56	43.00±2.56	44.00±2.04	46.00±2.31
	Female (n=5)	31.40±1.21	33.20±2.00	35.00±1.23	37.20±2.13	40.50±2.14
Group 4 1000\kg\body weight	Male (n=5)	40.00±2.78	42.00±1.38	45.00±2.61	46.00±2.61	48±2.47
	Female (n=5)	32.60±1.22	34.40±2.55	36.00±2.11	38.00±2.00	40.30±2.12

Values are expressed as mean ± SEM, n=5 females and 5 males

**Table 2:** effect of ethanol extract of *Psidium guajava* (EEPG) on organ weights in mice-sub-acute toxicity

	Treatment groups							
	**Control (1% CMC)**		**250 mg/kg**		**500 mg/kg**		**1000 mg/kg**	
	Males (n=5)	Females (n=5)	Males (n=5)	Females (n=5)	Males (n=5)	Females (n=5)	Males (n=5)	Females (n=5)
**Liver**	2.00 ± 0.44	1.45 ± 0.14	2.11 ± 0.24	1.88 ±0.14	2.15 ± 0.25	1.90 ± 0.25	2.26 ±0.44	1.85 ± 0.34
**Kidney**	0.50 ± 0.02	0.45 ± 0.08	0.48 ± 0.04	0.44 ± 0.06	0.50± 0.55	0.43 ± 0.11	0.46 ± 0.12	0.42 ±0.15
**Heart**	0.44 ± 0.02	0.42± 0.05	0.52±0.02	0.48±0.02	0.44±0.04	0.35±0.02	0.45±0.03	0.35±0.03

Values are expressed as mean ± SEM, n=5 females and 5 males; CMC: carboxymethyl cellulos

**Table 3:** effect of oral administration of the *Psidium guajava* extract on serum hematological parameters

Parameters								
**Female**				**Male**				
	**Control**	**250 mg/kg**	**500 mg/kg**	**1000 mg/kg**	**Control**	**250 mg/kg**	**500 mg/kg**	**1000 mg/kg**
WBC (103/μL)	5.00 ± 1.17	7.16 ± 1.63	5.66 ± 1.12	8.95 ± 2.00	5.40 ± 2.01	6.77 ± 1.10	8.00 ± 0.93	6.80 ± 2.26
Lymphocytes (%)	36.58 ± .03	36.25 ± 5.85	37.23 ± 0.97	38.85 ± 2.52	35.40 ± 5.65	38.42 ± 3.27	38.27 ± 3.87	39.66 ± 1.53
Monocyte (%)	8.77± 1.92	8.26 ± 1.65	7.63 ± 0.30	10.30 ± 2.43	9.36 ± 1.57	7.33 ± 0.76	9.22 ± 3.47	9.66 ± 3.55
Granulocytes (%)	9.66 ± 4.18	25.73 ± 9.08	21.40 ± 2.21	16.45 ± 3.00	22.20 ± 9.80	18.80 ± 6.90	20.47 ± 4.32	14.33 ± 1.90
PLT (103/μL)	677.3 ± 30.07	735.7 ±16.50	751.0 ±9.539	494.0 ± 76.08	396.0 ± 10.00	438.0 ± 47.84	529.7 ± 30.09	691.0 ± 108.5
MPV (fL)	11.83 ± 1.12	10.87 ± 1.79	10.93 ± 2.12	9.46 ±0.75	9.83 ±1.55	9.13 ± 0.37	8.80 ± 0.70	11.00 ± 1.40
RBC (106/μL)	7.29 ± 0.20	7.32 ± 0.45	7.05 ± 0.09	8.08 ± 0.24	6.34 ±1.93	8.07 ±0.30	7.89 ± 0.52	7.67 ± 0.24
HGB (g/dL)	16.20 ±0.10	16.40 ± 0.90	15.85 ± 0.05	16.10 ± 0.40	16.25 ± 0.05	17.07 ± 0.61	17.05 ± 0.15	17.00 ± 0.20
HCT (%)	43.50 ± 1.00	44.45 ± 2.45	41.50 ± 1.31	45.20 ± 2.10	43.25 ± 0.95	47.03 ± 0.80	45.53 ± 1.88	45.07 ± 4.53
MCV (fL)	62.60 ± 3.98	57.73 ± 2.80	58.93 ± 1.06	55.90 ± 3.89	58.00 ± 0.50	58.37 ± 1.41	60.15 ± 0.55	58.67 ± 4.10
MCH (pg)	22.70 ± 0.88	21.50 ± 0.50	21.87 ± 0.66	20.33 ± 1.42	21.75 ± 0.35	21.13 ± 1.29	21.20 ± 0.81	21.53 ± 0.80
MCHC g/dL)	37.20 ± 1.10	37.40 ± 0.95	37.37 ± 0.66	36.43 ± 1.65	37.55 ± 0.95	36.27 ± 1.80	36.63 ± 1.01	36.90 ± 3.57

Values are expressed as mean ± SEM, n=5 females and 5 males; WBC: white blood cells; PLT: platelets; MPV: mean platelet volume; RBC: red blood cells; HGB: hemoglobin; HCT: hematocrit; MCV: mean corpuscular volume; MCH: mean corpuscular hemoglobin; MCHC: mean corpuscular hemoglobin concentration

**Table 4:** effect of oral administration of the *Psidium guajava* extract on serum biochemical parameters

ALT (U/I)	50.20 ± 1.968	60.75 ± 2.404	70.36 ± 5.099	61.33 ± 1.758	89.57 ± 2.362	65.21 ± 3.148	35.49 ± 5.613	33.47 ± 3.573
AST (U/I)	33.27 ± 2.310	66.88 ± 1.817	56.02 ± 2.667	48.46 ± 5.535	67.44 ± 0.873	66.06 ± 2.197	69.84 ± 1.155b	65.85 ± 1.671
T. cholesterol (mg/dL)	55.14 ± 2.277	55.99 ± 2.364	59.27 ± 0.735	58.70 ± 1.767	92.69 ± 2.414	60.79 ± 4.614	56.19 ± 1.068	72.51 ± 1.258
HDL (mg/dL)	33.72 ± ± 0.705	31.88 ± 5.009	23.57 ± 1.301	20.04 ± 3.239	29.74 ± 2.260	40.24 ± 4.495	45.80 ± 4.061	50.92 ± 1.596
LDL (mg/dL)	20.25 ± 2.873	31.32 ± 7.160	40.58 ± 0.7714	33.99 ± 4.274	60.70 ± 0.777	30.84 ± 6.584	16.16 ± 4.669	22.42 ± 0.809
Triglycerides (mg/dL)	54.14 ± 0.885	51.02 ± 1.113	49.37 ± 0.692	46.71 ± 1.113	53.77 ± 0.692	51.48 ± 1.262	53.87 ± 1.301	54.14 ± 1.113
Creatinine (mg/dL)	1.22 ± 0.045	0.571 ± 0.054	0.72 ± 0.045	0.488 ± 0.023	0.55 ± 0.038	0.0.72 ± 0.045	0.9 ± 0.071	1.00 ± 0.086
Urea (mg/dL)	15.91 ± 4.243	20.41 ± 6.263	36.28 ± 1.389	25.26 ± 1.604	30.83 ± 1.389	26.07 ± 5.782	29.70 ± 2.122	36.81 ± 5.613
T. proteins (g/dL)	6.926 ± 0.071	7.192 ± 0.112	5.072 ± 0.124	6.501 ± 0.023	7.426 ± 0.141	6.401 ± ± 0.117	4.015 ± 0.069	5.878 ± 0.118

values are expressed as mean ± SEM, n=5 females and 5 males; AST: aspartate aminotransferase; ALT; alanine aminotransferase; LDL: low-density lipoprotein; HDL: high-density lipoprotein

**Figure 1:**
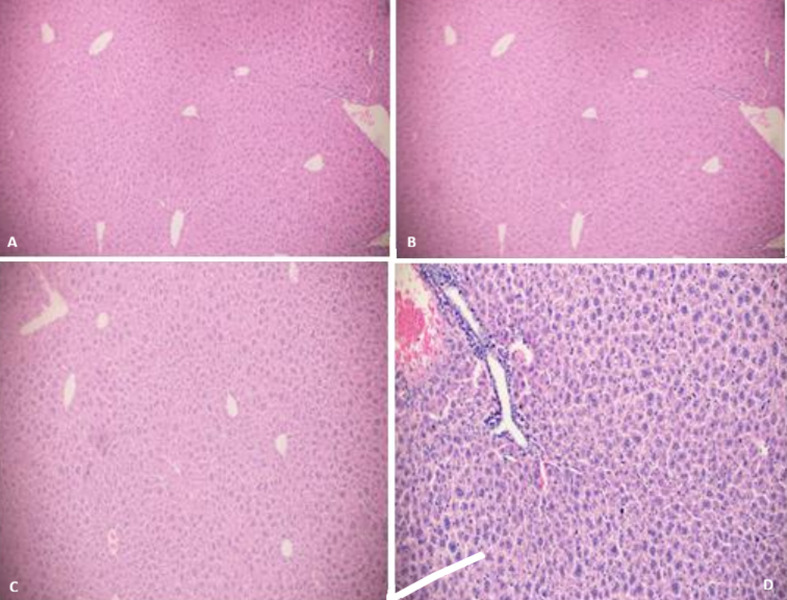
A,B,C,D) liver (L) sections of female mice showing effects of oral administration of the *Psidium guajava* extract over 28 days; L0) control group; L1)250 mg/kg; L2) 500 mg/kg; L3) 1000 mg/kg

**Histological studies:** to determine whether the daily administration of *P. guajava* ethanol leaf extract had harmed the kidney, liver, or heart of the animals, histopathology analyses were conducted; various findings are shown. At doses of 250 and 500 mg/kg b.w., the liver of females (Figure 1) showed no extract-related adverse effect; however, at 1000 mg/kg, there was inflammation (leucocyte infiltration). However, the kidney and heart did not exhibit any modifications. In addition, in male mice, no harm was observed to the kidney, liver, or heart at any of the assessed dose levels.

## Discussion

The growing reliance on herbal remedies, particularly in developing nations, is often supported by the perception that natural origin equates to inherent safety. This misconception leads to widespread self-medication without adequate consideration of potential health risks [16]. However, all bioactive compounds possess inherent toxic potential, making rigorous pre-clinical safety evaluation essential. Acute and sub-acute toxicity studies provide fundamental data on clinical signs, dose ranges, and therapeutic indices, which are critical for assessing the safety profile of any prospective pharmacologic agent [17]. The present study was therefore conducted to systematically evaluate the toxicological profile of a standardized *Psidium guajava* leaf ethanol extract (EEPG). In the acute toxicity study, no mortality or significant signs of morbidity were observed in mice administered a single high dose of 2000 mg/kg EEPG over the 14-day observation period. This indicates that the median lethal dose (LD_50_) for the extract is greater than 2000 mg/kg body weight. Based on this safety threshold, doses of 250, 500, and 1000 mg/kg/day (representing fractions of the LD_50_) were selected for the subsequent 28-day sub-acute study. Our work is similar to that of different scholars [18]. The repeated-dose study revealed no treatment-related mortality. Body weight gain and relative organ weights (for the heart, liver, kidneys, spleen, and lungs) showed no statistically significant differences between control and treatment groups. As changes in these parameters are sensitive indicators of systemic toxicity [19, 20], these results suggest an absence of adverse physiological or pathological effects. Furthermore, daily food and water consumption remained unaffected, indicating no disruption to basic metabolism or appetite [21,22].

Hematological analysis is a crucial component of toxicity assessment, as changes in blood parameters are highly informative for translational risk evaluation [23]. In this study, 28-day administration of EEPG did not alter key hematological markers (red blood cell (RBC), white blood cell (WBC), hemoglobin, platelet counts), confirming no adverse effect on hematopoietic function. Hepatic and renal functions were assessed through serum biochemistry. Levels of the liver enzymes ALT and AST, sensitive markers of hepatocellular injury [24], remained unchanged in all treatment groups. Similarly, bilirubin and total protein levels, indicators of liver synthetic function and biliary health [23], were unaltered. Renal function, assessed via blood urea nitrogen (BUN) and creatinine, also remained normal. This collective biochemical evidence strongly indicates that sub-acute EEPG administration did not induce hepatotoxicity or nephrotoxicity. The biochemical and hematological findings were further supported by histopathological examination. Microscopic analysis of vital organs (liver, kidney, heart) revealed no structural abnormalities, lesions, or signs of inflammation in any treatment group compared to controls in male animals, but some inflammation was observed in the liver of female mice. Integrating all endpoints-clinical observation, body and organ weights, hematology, serum biochemistry, and histopathology-this study demonstrates a notable absence of toxicity from EEPG at doses up to 1000 mg/kg/day over 28 days. Consequently, the no observed adverse effect level (NOAEL) for the extract is established as greater than 1000 mg/kg/day. These preclinical findings substantiate the safety of *Psidium guajava* leaf extract at the tested doses and support its traditional use. The extract can be classified as practically non-toxic. This safety profile provides an essential foundation for further research, including investigations into specific therapeutic mechanisms, chronic toxicity assessments, and ultimately, well-designed clinical trials to validate its efficacy and safety in humans.

## Conclusion

In the acute toxicity trials, treatment with single oral dosages of 2000 mg/kg did not cause any toxic symptoms or death. Over the course of 28 days, daily oral treatment of *Psidium guajava* ethanol extract did not result in changes in body weight, mortality, or weight increase. Additionally, at the conclusion of the experiment, no appreciable changes in hematological, biochemical, or histopathological abnormalities were noted. As a result, it was discovered that the extract's level of no harmful effects exceeded 1000 mg/kg/day/body weight. Overall, it can be said that for 28 days, the ethanol extract was well tolerated at a daily dosage of 1000 mg/kg.

### What is known about this topic

- Psidium guajava is a globally recognized medicinal plant with documented anti-diarrheal and hypoglycemic properties;
- Preliminary toxicity studies exist but often lack standardization and comprehensive organ function analysis;
- Phytochemical analyses revealed that the therapeutic effects of Psidium guajava extract are due to its rich content of bioactive compounds, including flavonoids, tannins, triterpenes, and essential oils.

### What this study adds

- This study provides the first organization for economic co-operation and development guideline-compliant toxicity assessment for Ethiopian P. guajava, featuring full hematological, biochemical, and histopathological evaluation;
- It establishes a clear dose-response relationship and identifies a specific NOAEL through a multi-dose sub-acute study, offering critical data for calculating safe human doses;
- And it contextualizes safety within traditional use, supporting the safe dosage range for its application in treating diarrheal diseases in Ethiopia.
